# Optimizing *Artemia* Enrichment: A Low DHA/High EPA Protocol for Enhanced *n*-3 LC-HUFA Levels to Support Greater Amberjack (*Seriola dumerili*) Larval Rearing

**DOI:** 10.1155/2023/5548991

**Published:** 2023-09-19

**Authors:** Javier Roo, Daniel Montero, Quirós-Pozo Raquel, Christian Monzón-Rivero, Marisol Izquierdo López

**Affiliations:** Aquaculture Research Group (GIA), Institute of Sustainable Aquaculture and Marine Ecosystems (IU-ECOAQUA), Universidad de Las Palmas de Gran Canaria (ULPGC), Carretera de Taliarte, s/n, Telde 35214, Spain

## Abstract

Most commercial products available for *Artemia* sp. enrichment include high levels of docosahexaenoic acid (DHA) to boost its nutritional value, though with limited success. In this regard, the present study evaluated the alternative utilization of eicosapentaenoic acid (20 : 5*n*-3; EPA) oils to improve the *n*-3 long-chain highly unsaturated fatty acid content (*n*-3 LC-HUFA) in enriched *Artemia* sp. to feed greater amberjack (*Seriola dumerili*) larvae. Five experimental emulsions containing increasing levels of EPA from 0.8% to 60% of total fatty acids (TFA) and *n*-3 LC-HUFA (1.3%–70.6% TFA) were formulated. Each diet was fed to greater amberjack larvae (17–35 days posthatch (dph)) in three replicate 200-L tanks. The dietary EPA supplementation significantly improved larval growth during the feeding trial and survival at 35 dph (*P* < 0.05). In addition, larval fatty acid profiles showed a positive correlation with dietary EPA. Finally, despite the sum of total skeletal anomalies and column anomalies were not significantly affected by dietary EPA, increasing dietary EPA, and *n*-3 LC-HUFA tended to reduce the incidence of these types of anomalies in greater amberjack larvae at 35 dph. Based on these results, *S. dumerili* larvae could be successfully grown with low DHA but high EPA-rich oil enrichment products when *n*-3 LC-HUFA content in *Artemia* sp. is maintained in sufficient amounts.

## 1. Introduction

The greater amberjack *Seriola dumerili* (Risso, 1810) is an excellent candidate for marine fish species aquaculture diversification due to its high consumer acceptability worldwide, high market value, and excellent flesh quality [1–3]. Although the aquaculture of *Seriola* sp. species has increased in recent years from 160,000 metric tons in 2015 to 167,000 metric tons in 2019 [4], their production is still constrained by the reduced number of commercial hatcheries and juveniles availability. Seriola juveniles for on-growing operations are mostly collected from the wild in Asian countries, while are produced in hatcheries in Europe, Australia, and America. In this regard, the limited knowledge of zootechnics and nutritional requirements during larval stages is one of the major causes for the poor hatchery survival and juvenile quality obtained under aquaculture sustainable methodologies. Up to date, the most common feeding protocol for greater amberjack larvae produced in hatcheries is based on the use of enriched rotifers (*Brachionus* sp.) from the first feeding, followed by the use of brine shrimp (*Artemia* sp. nauplii and meta nauplii) appropriately enriched with commercial or experimental products to finally cofeed during the weaning stages until complete replacement of live feed at later stages [5–11]. Thus, hatchery-reared juveniles are usually characterized by the high number of weak fish with low resistance to manipulation at early stages [10], high size variability, and high incidence of skeleton anomalies in comparison to the wild-captured ones [11]. Although some attempts are still underway to introduce the use of marine copepods as food for the first feeding of greater amberjack, the complexity and costs of copepod production limit this approach to a reduced number of commercial and experimental stations with specific knowledge and capacity to produce copepods at a large scale. Nevertheless, with an adequate formulation of the enrichment products and the knowledge and quantification of the species-specific requirements, standard live preys (rotifers and *Artemia* sp.) can be used as effectively as copepods for greater amberjack culture. One of the most important nutritional differences and benefits of copepods in comparison to rotifers and *Artemia* sp. is related to their optimal lipid classes and fatty acid profiles, which usually cover the nutritional needs of most marine fish larvae [12, 13]. In this regard, it is well known that one of the most important factors affecting the performance of marine fish larvae is the *n*-3 long-chain highly unsaturated fatty acid content (*n*-3 LC-HUFA) composition of their feed [14]. Thus, depending on the enzymatic capacity of the different species for desaturation and elongation to convert the C:18 polyunsaturated fatty acids, *α*-linolenic acid (18 : 3*n*–3), into the biologically active long-chain (C:20–24) polyunsaturated fatty acids, they must be included or not in the larval diets to fulfill their nutritional requirements [14]. Specifically, docosahexaenoic acid (DHA, 22 : 6*n*-3) and eicosapentaenoic acid (EPA, 20 : 5*n*-3) are essential components of cellular membranes, modulate physiological processes, including membrane transport, receptors, and enzymatic activities [15], and play essential roles in fish larval development in many marine species [10, 16–18]. However, to date, a limited number of studies have addressed the fatty acids requirements for greater amberjack during live feed stages, mostly focusing on DHA enrichment [8, 10]. As stated above, the feeding sequence of *S. dumerili* larvae usually includes *Artemia* sp. nauplii, enriched or not, from as early stages as 10 days posthatch (dph) [10, 11]. However, the fatty acid composition of *Artemia* sp., even after enrichment, is usually deficient in *n*-3 LC-HUFA, particularly in DHA [19, 20]. Major causes of this effect are associated with enrichment protocols themselves, that is, accelerating autoxidation of *n*-3 LC-HUFA due to high water temperatures (28°C) and vigorous aeration [21], while others are intrinsic to *Artemia* sp. metabolism, such as undesired metabolic retroconversion of DHA to EPA, its mobilization to neutral lipid fraction and major accumulation as triglycerides [20, 22].

In this regard, most of the commercial products available for *Artemia* sp. enrichment include high levels of DHA, trying to boost *n*-3 LC-HUFA and particularly DHA in *Artemia* sp. while maintaining a high (>2) DHA/EPA ratio. With this perspective, the objective of the present study was to determine the potential utilization of purified EPA oils as a substitute for DHA during *Artemia* sp. enrichment to cover the nutritional requirements of *n*-3 LC-HUFA in *S. dumerili* larvae, evaluating its effects on survival, growth, stress resistance/tolerance, skeletal anomalies development, and tissue composition.

## 2. Materials and Methods

### 2.1. Ethic Statement

All experimental protocols described in this manuscript fully comply with the recommendations in the Guide for Care and Use of Laboratory Animals of the European Union Council (2010/63/EU) and Spanish National Legislation (Spanish Royal Decree 53/2013) which regulate animal usage in experimentation and/or other scientific purposes, and with the general guidelines approved in the EU-project DIVERSIFY (KBBE.2013.1.2–09 KBBE.2013.1.2–09, grant agreement no. 603121).

### 2.2. Larval Rearing

Greater amberjack larvae of 17 dph, (*n* = 1,000 per tank; total length (TL) 6.39 ± 0.44 mm; fresh weight 2.94 ± 0.57 mg; mean ± standard deviation) were randomly distributed into 15 light gray, cylindrical fiberglass tanks (five triplicate treatments) of 200 L. All tanks were equipped with continuous aeration and supplied with filtered UV-sterilized seawater (salinity 37 psu) in a flow-through system. Water exchange was gradually increased from 0.80 L min^−1^ at 17 dph to 1.60 L min^−1^ at 25 dph and finally to 3.30 L min^−1^ from 30 dph to the end of the feeding trial at 35 dph, ensuring good water quality and removal of nonfeeding preys from 1 day to the next. The average seawater temperature and dissolved oxygen during the experimental period were 24.15 ± 0.35°C and 6.55 ± 0.41 ppm, respectively. An artificial fluorescent light above each tank provided a surface light intensity ranging between 1,000 and 1,500 lux (Digital Lux Tester YF-1065; Powertech Rentals, Osborne, Australia) at the center of each rearing tank, with a photoperiod of 12 hr light/12 hr darkness cycle with lights on from 08:30 a.m. to 8:30 p.m. From 17 to 22 dph, there was an overlap between rotifers (nonenrich) and enriched *Artemia* sp. with a gradual reduction in the amounts of nonenriched rotifers from 5,000 to 0 individuals L^−1^ and a progressive increasing density of enriched *Artemia* sp. (125–500 individuals L^−1^) provided two times per day (9:00–15:00 hr) during the same period. From 23 to 35 dph, larvae were exclusively fed with 500 individuals L^−1^ of enriched *Artemia* sp. two times per day (9:00–15:00 hr), which were offered following complete clearing of the tank between feedings.

### 2.3. Growth and Survival

All larval samplings were carried out randomly from each experimental tank 1 hr before the first feed at 17 dph and after night starvation at 35 dph. Larvae were euthanized with an overdose of clove oil containing 87% eugenol (Guinama®, Valencia, Spain) and TL was measured with a profile projector (Mitutoyo PJ-A3000, Kanagawa, Japan). Additionally, the fresh body weight (FW) of 30 larvae/tank was determined initially (17 dph) and finally at 35 dph. Larval survival was determined by hand counting of all the remaining alive larvae in each tank at the end of the experiment at 35 dph.

### 2.4. Stress Test

At 35 dph, 30 larvae per tank were used to conduct an acute air stress test, by handling them out of the water in a scoop net for 30 s. This test was repeated with another 30 larvae, but increasing the exposure time to 60 s. After the test, each set of larvae was returned to separate buckets with aerated seawater to determine survival after 24 hr [10, 23].

### 2.5. Experimental Enrichments

#### 2.5.1. Preparation

To determine the capacity of EPA inclusion in enrichments to raise the *n*-3 LC-HUFA content in *Artemia* sp., five experimental enrichments (EPA 0–4) with increasing EPA content (0%–60%) were formulated (Table 1). The enrichments were prepared by mixing increasing amounts of a commercial oil (Incromega EPA 500TG; Croda, Barcelona, Spain) containing 63% EPA and 8% DHA in total fatty acids (TFA) with a second commercial oil (Oleic Oil; Sigma–Aldrich, Madrid, Spain) containing 77% oleic acid (OA, 18 : 1*n*-9) in TFA. Additionally, every enrichment preparation was mixed with constant amounts of soya lecithin (Korot SL, Alcoy, Spain) containing 54% linoleic acid (18 : 2*n*-6) and trace amounts of EPA and DHA as emulsifier. Also, enrichment preparations were fortified with 3 g kg^−1^ vitamin E (DL-*α*-tocopherol acetate; Sigma–Aldrich, Madrid, Spain) and 2.5 g kg^−1^ vitamin C (L-ascorbic acid, Asc; Sigma–Aldrich, Madrid, Spain) to prevent the oxidation according to Betancor et al. [24], Hamre et al. [13], and Roo et al. [10]. Once prepared, experimental enrichments were stored in a fridge at 4°C until used. During the feeding trial, three subsamples of each experimental enrichment (ca. 100 g), newly hatched *Artemia* sp. (ca. 100 g), and enriched *Artemia* sp. (ca. 100 g) were collected and analyzed for proximate and fatty acid composition (Table 2).

#### 2.5.2. Artemia sp. Enrichment

Brine shrimp *Artemia* sp. cysts (Sept Art type; INVE Aquaculture, Dendermonde, Belgium) were incubated for 24 hr in 150 L cylindrical–conical tanks with highly aerated seawater (37 g L^−1^) at 28°C. Newly hatched nauplii were harvested, washed, and transferred to a 10 L bucket (one bucket for each experimental emulsion) filled with clean seawater and provided with moderated aeration, oxygen supply automatically controlled to maintain 6.0 ± 0.2 ppm and 28°C temperature. To enrich the *Artemia* sp., 1.1 ml of each experimental enrichment was stirred for 1 min with 100 ml of fresh water and added to each biker. The same enrichment conditions were utilized for all the experimental enrichments assayed, including enrichment time (18 hr), *Artemia* sp. density (500,000 individuals L^−1^) and amount of experimental enrichment.

### 2.6. Lipid Content and Fatty Acid Analysis

A sample of larvae (ca. 50 individuals) from each experimental tank at 35 dph was collected for biochemical analysis. All samples were flushed with N_2_ and kept frozen at −80°C until analysis was carried out. Total lipids were extracted in chloroform : methanol (2 : 1, v : v) using the method of Folch et al. [25]. Fatty acids were prepared by transesterification using the method of Christie and Han [26]. Separation and identification of the fatty acids was realized with gas chromatography (GC Thermo Finnigan Fucus GC, Milan, Italy) under the conditions reported by Izquierdo et al. [27]. Dry matter, lipids, and ash content were determined using the methods of analysis of the Association of Official Analytical Chemists [28]. Results of total lipids, TFA content in the enrichment emulsions, enriched *Artemia* sp. and larvae for each treatment are shown in Tables 1–3, respectively. Data are expressed as percentage of total fatty acids (% TFA).

### 2.7. Skeletal Anomalies

At 35 dph, 100 larvae per tank were euthanized with clove oil and fixed in 10% buffered formaldehyde, cleared, stained with alizarin red, and immediately photographed to evaluate skeletal anomalies occurrence [29]. Anomalies were studied in the different axial column regions according to the methods described by Boglione et al. [30].

### 2.8. Statistics

All the data were statistically analyzed using SPSS Statistical Software System 15.0 (SPSS, http://www.spss.com). Data were presented as mean + standard deviation. Effects of the dietary levels of EPA were analyzed by regression and analysis of variance at a significant level of 5%. All variables were checked for normality and homogeneity of variance using the Kolmogorov–Smirnoff and the Levene tests, respectively [31].

The nonparametric Kruskal–Wallis test was accomplished in the analysis of survival after stress test data as a result of heterogeneous variance. To evaluate the differences in skeletal anomalies, logarithmic linear statistical analysis was performed [31].

## 3. Results

### 3.1. Experimental Emulsions and Artemia sp. Enrichment

Formulations and FA compositions of the experimental emulsions (EPA 0–4) are listed in Table 1. The use of OA oil to compensate for the reduction of EPA-rich oil (EPA 500TG) resulted in variations in saturated, monoenoic, total *n*-3, total *n*-3 LC-HUFA, total *n*-9, and different FA ratios such as DHA/EPA between the experimental emulsions. The lowest level of EPA in the experimental emulsions was present in EPA-0 with 0.8% in TFA, while the EPA content in EPA 1–4 increased from 14.2% to 60.1% in TFA. The DHA and arachidonic acid (ARA) in the experimental emulsions increased in accordance with EPA, from 0.3% to 6.7% and from 0% to 3.5% in TFA, respectively. *Artemia* sp. enriched with different experimental emulsions resulted in five enriched *Artemia* sp. treatments (Table 2). Thus, EPA and *n*-3 LC-HUFA content in *Artemia* sp. mirrored EPA and *n*-3 LC-HUFA content in the experimental emulsion, ranging from 1.08% to 22.9% and from 3.2% to 29.0%, respectively. Other fatty acids such as DHA (ranged from 0.14% to 3.01% TFA) and ARA (ranged from 0.39% to 1.72% TFA) were also correlated with their emulsion content. On the other hand, the DHA/EPA ratio was kept quite constant (average 0.1) for all the experimental treatments (Table 2).

### 3.2. Larval Growth and Survival

**Figure 1:**
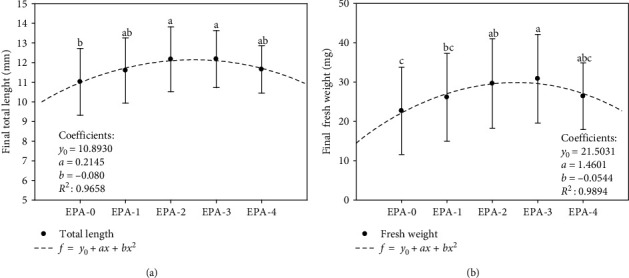
Relationship between (a) total length (mm) and (b) fresh weight (mg) to dietary EPA (20 : 5*n*-3) content (% TFA) in larval greater amberjack at 35 dph (mean + *SD*, *n* = 3). Data are fitted to a quadratic regression analysis (*f* = *y*_0_ + *ax* + *bx*^2^).

**Figure 2:**
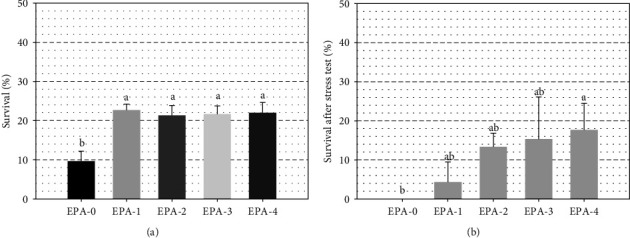
(a) Final survival and (b) survival after 30 s stress test at 35 dph greater amberjack larvae, fed different dietary. EPA (20 : 5*n*-3) content (% TFA). Values are mean + *SD*, *n* = 3.

Larval growth was significantly improved with the increase of dietary EPA and *n*-3 LC-HUFA during the feeding trial (Figures 1(a) and 1(b)). Mean values for all the dietary treatments were 11.72 ± 0.47 mm in TL and 27.13 ± 3.21 mg in FW at 35 dph. Larval fed EPA-2 and EPA-3 showed significant (*P* < 0.05) better growth in terms of TL than those larvae fed EPA-0, but similar to EPA-1 and EPA-4 (Figure 1(a)). Growth in terms of fresh weight followed a similar pattern to that in TL. Thus, larvae fed EPA-3 showed significantly (*P* < 0.05) higher FW than EPA-0 and EPA-1 but like EPA-2, and EPA-4 (*P* > 0.05) (Figure 1(b)). The relationship between final TL and *Artemia* sp. EPA content was described by the second-order polynomial regression: *y* = 10.893 + 0.214*x* − 0.080 *x*^*2*^ (*y* = Total length; *x* = Dietary EPA (% TFA); *R*^2^ = 0.965; *P* ≤ 0.05). Also, the final fresh weight was described by the equation: *y* = 21.503 + 1.460*x* – 0.0544 *x*^*2*^ (*y* = Fresh weight; *x* = Dietary EPA (% TFA); *R*^2^ = 0.989; *P* ≤ 0.05). Under the experimental conditions applied, both growth models suggest that maximum growth would be achieved in the range of dietary EPA and *n*-3 LC-HUFA concentrations tested, between 8%–16% TFA and 11%–21% TFA, respectively. Specifically, the highest growth values would be achieved at 13.3% EPA and 17.7% *n*-3 LC-HUFA content in *Artemia* sp. Larval survival was significantly (*P* ≤ 0.05) improved by dietary EPA at 35 dph, the lowest survival was recorded in those larvae receiving the lowest EPA and *n*-3 LC-HUFA in the *Artemia* sp. (EPA-0). Hence, larval resistance to stress test (30 s), determined as the survival rate 24 hr after handling, was significantly affected by dietary EPA and *n*-3 LC-HUFA at 35 dph, being the lowest survival recorded in those larvae fed the lowest EPA and *n*-3 LC-HUFA in the diet (EPA-0) (Figures 2(a) and 2(b)), but no survivors were recorded at 60 s stress test in any treatment assayed.

### 3.3. Proximate and Fatty Acid Composition in S. dumerili Larvae

The results of the total lipid and fatty acid composition of *S. dumerili* larvae at 35 dph are shown in Table 3. Total lipids, ash content, and moisture were similar for the larvae fed any of the *Artemia* sp. treatments. Regarding the larvae FA composition, *n*-3 LC-HUFA were significantly (*P* < 0.05) affected by increased levels of dietary EPA in EPA-0 and EPA-1 treatment in comparison to EPA-4. Similarly, the EPA level in larvae fed EPA-0 was significantly (*P* < 0.05) different than EPA-4 fed ones. DHA contents in larval tissues were also affected by dietary treatments, with the lowest values for those larvae fed EPA-0, which was significantly (*P* < 0.05) lower than those larvae fed EPA-4. On the other hand, ARA content or DHA/EPA ratios in larval tissues were not affected (*P* > 0.05) by the different treatments.

### 3.4. Larval Quality

**Figure 3:**
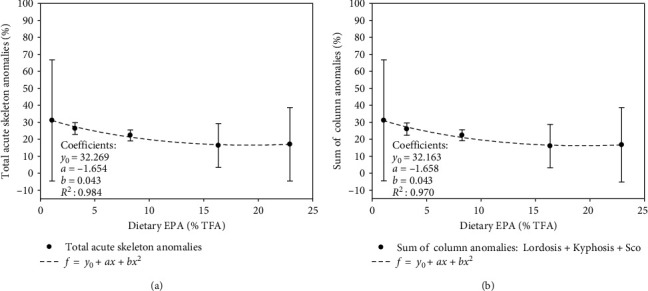
Relationship between (a) total acute skeleton anomalies (%) and (b) sum of column anomalies to dietary EPA (20 : 5*n*-3) content (% TFA) in greater amberjack larvae at 35 dph (mean + *SD*, *n* = 3). Data are fitted to a quadratic regression analysis (*f* = *y*_0_ + *ax* + *bx*^2^).

The incidence of total acute skeleton anomalies was not significantly affected by dietary EPA and *n*-3 LC-HUFA with an average value for all dietary treatments of 22.63% ± 6.25% (Figure 3(a)). Regardless of the EPA and *n*-3 LC-HUFA content, most of the acute skeleton anomalies were major alterations in the hemal region, related to column anomalies, with scoliosis being the most important recorded affection (Figure 3(b)). The relationship between total acute skeleton anomalies and *Artemia* sp. EPA content was described by the second-order polynomial regression: *y* = 32.269 + (−1.654*x*) + 0.043*x*^*2*^ (*y* = Total anomalies; *x* = Dietary EPA (% TFA); *R*^2^ = 0.9854; *P* ≤ 0.05) (Figure 3(a)). Similarly, the relationship between the sum of column anomalies and *Artemia* sp. EPA content was described by the same model, represented as: *y* = 32.163 + (−1.658*x*) + 0.043*x*^*2*^ (*y* = Total column anomalies; *x* = Dietary EPA (% TFA); *R*^2^ = 0.9854; *P* ≤ 0.05) (Figure 3(b)), suggesting that increasing dietary EPA and *n*-3 LC-HUFA tends to reduce the incidence of these anomalies during the live stage of development evaluated. Under the experimental conditions and the regression models applied, these results suggest that the best larval quality in terms of skeleton anomalies occurrence was achieved at EPA levels between 16% and 21% TFA with a maximum around 17% EPA content in *Artemia* sp., which matched with the data obtained to reach the best growth.

## 4. Discussion

Present results demonstrated successful EPA and *n*-3 LC-HUFA enrichment of *Artemia* sp. nauplii with the experimental emulsions formulated. Gradual increase in EPA and *n*-3 LC-HUFA levels in *Artemia* sp. improved greater amberjack larval growth. Thus, the highest growth was obtained when larvae were fed *Artemia* sp. containing *n*-3 LC-HUFA in a range of 11%–21% TFA (2.3–5.3 g 100 g^−1^*n*-3 LC-HUFA dry weight (dw)), with EPA content between 8% and 16% TFA (0.8–2.2 g 100 g^−1^ EPA dw) despite a low DHA level (0.87%–2.22% TFA; 0.1–0.5 g 100 g^−1^ DHA dw) and a low DHA/EPA ratio (average 0.1). Furthermore, the optimal *n*-3 LC-HUFA levels based on EPA provision in this study is consistent with the optimum dietary *n*-3 LC-HUFA levels determined to obtain maximum growth and survival in a previous study conducted by our team but using rich DHA oils as the main *n*-3 LC-HUFA source [10] These data also agreed with *n*-3 LC-HUFA levels reported by Takeuchi et al. [32] and Ishizaki et al. [33] in other carangid species such as striped jack (*Pseudocaranx dentex*) and yellowtail (*Seriola quinqueradiata*). These authors did not find significant differences in either growth or survival when enriched *Artemia* sp. was used with both DHA-rich oil or EPA ones with *n*-3 LC-HUFA levels in the range of 11%–25% TFA and 13%–21% TFA, respectively. Hence, Matsunari et al. [7] reported better growth and survival in younger amberjack larvae (3–10 dph) when *n*-3 LC-HUFAs are in the range of 15%–22% TFA but using DHA-rich oils. In the same way, present data showed a reduction in greater amberjack larval growth and survival when fed on *Artemia* sp. enriched with *n*-3 LC-HUFA content lower than 11% TFA. In the aforementioned trials with greater amberjack, yellowtail, and striped jack, all of those species performed significantly better when fed live preys containing *n*-3 LC-HUFA levels over this value, which could be considered as a minimum amount to be raised during live prey enrichment for greater amberjack larval production [10]. Additionally, the negative effect on larval growth when the level of *n*-3 LC-HUFA is higher than 21% might be an indication of an excess of *n*-3 LC-HUFA beyond larval requirements. Conversely, increased levels of DHA and EPA and DHA/EPA ratios in live preys are reported as nutritional indicators to promote marine fish larval growth, survival, stress resistance, pigmentation, and larval quality [15, 34, 35]. Thus, target values of DHA/EPA over 1.0 and even close to 2.0 or higher are usually recommended during live prey enrichment, taking as reference values reported in natural live preys such as copepods [12]. However, the intrinsic capacity of *Artemia* sp. to metabolize DHA during the enrichment and postenrichment process, led to the retroconversion of DHA to EPA [19, 22], thus reducing the DHA/EPA ratio and the nutritional value of this live prey. In this study, the utilization of EPA-rich oil, as a main source of *n*-3 LC-HUFA, despite elevating the *n*-3 LC-HUFA content in *Artemia* sp., led to a reduction in DHA/EPA ratio to values close to 0.1, but with an efficient utilization of the fatty acids provided in the experimental emulsion, with no apparently harmful effects on larval growth, survival, resistance to stress test or quality when sufficient *n*-3 LC-HUFA were provided. Most published studies report that DHA is more limiting for growth, survival, and nervous system and schooling behavior development than EPA [15, 36]. On the contrary, other studies identify high levels of dietary DHA as a cause of muscular dystrophy [37] or spinal anomalies in larvae [38] due to the high peroxidation risk of DHA and the formation of toxic oxidized compounds, if they are not properly protected with antioxidant substances [18, 35].

In this regard, the relationship between dietary *n*-3 LC-HUFA and its role in bone growth and development has been previously addressed [17, 35]. Present data showed a 10% reduction in total acute and sum of column skeleton anomalies when *n*-3 LC-HUFA content was increased from 3% to 11% TFA, suggesting the beneficial effect of *n*-3 LC-HUFA supplementation and confirming the appropriated FA antioxidant protection during experimental enrichment formulation.

## 5. Conclusion

Based on these results, the utilization of EPA-rich oils to increase *n*-3 LC-HUFA content in *Artemia* sp. seems to be an effective option to improve *Artemia* sp. nutritional quality. Additionally, the utilization of this experimental enrichment with a *n*-3 LC-HUFA content between 11% and 21% TFA and current methodology avoids *Artemia* sp. nauplii retroconversion of DHA to EPA, providing a sufficient amount of *n*-3 LC-HUFA for greater amberjack larvae during *Artemia* sp. feeding stage, with no harmful effect over larval growth, survival, and stress resistance.

## Figures and Tables

**Table 1:** Experimental emulsion ingredients (g kg^−1^) and selected fatty acid contents (percentage of total fatty acids, % TFA) of total lipids in the experimental emulsions.

	EPA-0	EPA-1			EPA-2	EPA-3			EPA-4
Ingredients (g kg^−1^ diet)									
Incromega EPA 500TG	0	300			450	600			900
Oleic acid (OA)	900	600			450	300			0
Soybean lecithin	94.5	94.5			94.5	94.5			94.5
DL-*α*-tocopherol acetate	3	3			3	3			3
Vitamin C	2.5	2.5			2.5	2.5			2.5
Fatty acid content (% TFA)									
Saturated	15.21	12.66			11.46	9.27			2.61
Monoenoics	74.63	58.63			50.00	36.95			7.60
*n*-3	2.16	20.48			30.11	45.09			79.52
*n*-6	7.93	7.43			7.16	6.95			7.65
*n*-9	72.17	56.07			47.47	34.57			5.56
Total *n*-3 LC-HUFA	1.37	17.35			25.60	38.74			70.62
14 : 0	0.02	0.12			0.20	0.27			0.35
16 : 0	11.45	9.21			7.99	6.01			0.95
16 : 1*n*-7	0.76	0.81			0.88	0.89			0.87
18 : 0	3.14	2.54			2.25	1.72			0.41
18 : 1*n*-9 (OA)	72.13	55.84			47.13	34.06			3.94
18 : 1*n*-7	1.40	1.46			1.33	1.16			0.94
18 : 2*n*-6 (LA)	7.80	6.14			5.16	4.05			1.96
18 : 3*n*-3 (ALA)	0.67	0.65			0.64	0.66			1.18
20 : 1*n*-9	0.02	0.01			0.01	0.02			0.41
20 : 4*n*-6 (ARA)	0.06	0.82			1.25	1.83			3.53
20 : 5*n*-3 (EPA)	0.84	14.28			21.18	31.97			60.16
22 : 6*n*-3 (DHA)	0.30	2.36			3.34	5.11			6.79
ARA/EPA	0.07	0.06			0.06	0.06			0.06
DHA/EPA	0.36	0.17			0.16	0.16			0.11
DHA/ARA	5.41	2.89			2.68	2.80			1.92
OA/DHA	240.20	23.67			14.13	6.66			0.58
OA/*n*-3 LC-HUFA	52.71	3.22			1.84	0.88			0.06
*n*-3/*n*-6	0.27	2.76			4.21	6.49			10.40

**Table 2:** Proximate (% dry matter) and fatty acids composition (% TFA) of initial *A. salina* nauplii and *A. salina* nauplii after 18 hr enrichment with five experimental emulsions.

	*Artemia nauplii*	EPA-0	EPA-1	EPA-2	EPA-3	EPA-4
Proximate analysis (% dry matter)						
Lipids	16.46 ± 0.80	19.72 ± 1.04	19.45 ± 1.97	24.29 ± 3.16	25.36 ± 1.06	26.31 ± 0.68
Moisture	91.01 ± 0.04	90.33 ± 0.21	90.39 ± 0.21	90.05 ± 0.35	90.00 ± 0.30	89.05 ± 0.12
Ash	9.53 ± 0.63	10.79 ± 0.61	10.16 ± 0.43	10.09 ± 0.69	8.61 ± 1.49	9.76 ± 048
Fatty acid content (% TFA)						
Saturated	19.64	19.07^a^	19.93^a^	16.18^ab^	12.56^bc^	10.21^c^
Monoenoics	30.64	41.31^a^	40.38^a^	37.26^a^	30.28^b^	22.44^c^
*n*-3	39.59	29.51^b^	29.64^b^	36.16^b^	46.74^a^	55.40^a^
*n*-6	7.59	7.76^b^	7.56^b^	8.00^b^	7.92^b^	8.87^a^
*n*-9	19.66	31.80^a^	30.08^a^	28.51^a^	23.14^b^	15.06^c^
Total *n*-3 LC-HUFA	4.55	3.24^d^	5.33^d^	11.34^c^	21.16^b^	29.07^a^
14 : 0	0.53	0.46^ab^	0.57^a^	0.40^b^	0.32^b^	0.34^b^
16 : 0	10.32	10.66^a^	11.11^a^	8.83^ab^	6.84^b^	6.90^b^
16 : 1*n*-7	1.95	1.75^ab^	1.93^a^	1.63^ab^	1.40^b^	1.50^b^
18 : 0	7.63	6.86^a^	7.15^a^	6.00^a^	4.66^ab^	2.23^b^
18 : 1*n*-9 (OA)	18.41	30.70^a^	29.00^a^	27.44^a^	22.07^b^	13.77^c^
18 : 1*n*-7	7.63	6.39^ab^	6.83^a^	5.92^b^	4.83^c^	4.97^c^
18 : 2*n*-6 (LA)	5.70	6.30^a^	6.06^a^	6.10^a^	5.53^b^	5.70^b^
18 : 3*n*-3 (ALA)	29.64	23.03	21.61	21.66	21.56	21.80
20 : 1*n*-9	0.03	0.03^a^	0.03^a^	0.03^a^	0.03^a^	0.09^b^
20 : 4*n*-6 (ARA)	0.56	0.39^d^	0.51^d^	0.81^c^	1.23^b^	1.72^a^
20 : 5*n*-3 (EPA)	1.81	1.08^e^	3.18^d^	8.26^c^	16.34^b^	22.91^a^
22 : 6*n*-3 (DHA)	0.08	0.14^d^	0.27^d^	0.87^c^	2.22^b^	3.01^a^
ARA/EPA	0.31	0.38^a^	0.17^b^	0.11^bc^	0.07^c^	0.07^c^
DHA/EPA	0.04	0.13^ab^	0.09^c^	0.10^bc^	0.14^a^	0.13^a^
DHA/ARA	0.14	0.35^c^	0.53^c^	1.02^b^	1.81^a^	1.76^a^
OA/DHA	238.31	253.82^a^	115.64^b^	38.41^b^	9.98^b^	4.58^b^
OA/*n*-3 LC-HUFA	4.05	9.87^a^	6.22^ab^	2.74^bc^	1.04^c^	0.47^c^
*n*-3/*n*-6	5.22	3.79^b^	3.90^b^	4.52^b^	5.90^a^	6.24^a^

*Note*: Proximate analysis data represent means ± SD. (*n* = 3). FA content data are presented as mean. Different superscripts within each row indicate a significant difference between diets (ANOVA (*P* ≤ 0.05); Tukey's HSD). HUFA, highly unsaturated fatty acid; ARA, arachidonic acid; DHA, docosahexaenoic acid; EPA, eicosapentaenoic acid.

**Table 3:** Proximate (lipid, moisture, and ash content, % dry matter) and fatty acids composition (% TFA) of *S. dumerili* larvae 35 dph fed enriched *Artemia* with different experimental emulsions.

	EPA-0	EPA-1	EPA-2	EPA-3	EPA-4
Proximate analysis (% dry matter)					
Lipids	17.27 ± 3.85	14.71 ± 1.70	16.08 ± 1.04	15.00 ± 1.75	16.10 ± 1.39
Moisture	84.58 ± 2.35	86.87 ± 1.10	87.24 ± 3.66	85.59 ± 0.70	87.71 ± 1.12
Ash	15.99 ± 0.02	16.43 ± 1.41	16.03 ± 1.49	15.95 ± 2.40	15.19 ± 0.93
Fatty acid content (% TFA)					
Saturated	27.00 ± 1.30^ab^	28.93 ± 1.71^a^	27.61 ± 0.59^ab^	24.11 ± 0.82^c^	24.93 ± 0.27^bc^
Monoenoics	29.07 ± 3.23^a^	28.75 ± 0.81^a^	28.61 ± 0.27^a^	27.98 ± 0.37^a^	23.00 ± 0.24^b^
*n*-3	28.78 ± 5.25^b^	27.83 ± 0.56^b^	30.31 ± 0.21^b^	33.37 ± 2.16^ab^	38.68 ± 0.42^a^
*n*-6	11.62 ± 1.08	11.02 ± 0.43	10.28 ± 0.18	10.01 ± 0.79	9.97 ± 0.23
*n*-9	21.60 ± 3.07^b^	21.58 ± 0.60^b^	21.45 ± 0.22^b^	20.01 ± 1.56^ab^	15.84 ± 0.25^b^
Total *n*-3 LC-HUFA	15.59 ± 6.24^b^	17.31 ± 0.59^b^	20.09 ± 0.23^ab^	21.84 ± 0.91^ab^	26.31 ± 0.26^a^
14 : 0	0.51 ± 0.16	0.40 ± 0.15	0.31 ± 0.02	0.36 ± 0.09	0.35 ± 0.03
16 : 0	14.22 ± 0.32^ab^	14.60 ± 0.36^a^	14.36 ± 0.20^ab^	12.50 ± 0.83^c^	13.24 ± 0.23^cb^
16 : 1*n*-7	0.57 ± 0.04	0.54 ± 0.01	0.54 ± 0.00	0.76 ± 0.36	0.57 ± 0.01
18 : 0	11.37 ± 1.08^ab^	13.13 ± 1.81^a^	12.13 ± 0.44^ab^	10.16 ± 0.55^b^	10.59 ± 0.11^ab^
18 : 1*n*-9 (OA)	18.83 ± 3.29^a^	19.16 ± 0.41^a^	19.28 ± 0.18^a^	17.23 ± 2.56^ab^	13.50 ± 0.22^b^
18 : 1*n*-7	6.11 ± 0.32	5.84 ± 0.14	5.85 ± 0.06	5.76 ± 0.37	5.80 ± 0.02
18 : 2*n*-6 (LA)	6.55 ± 0.71^a^	5.82 ± 0.23^ab^	5.35 ± 0.14^b^	4.79 ± 0.43^b^	5.05 ± 0.32^b^
18 : 3*n*-3 (ALA)	11.25 ± 1.27	8.99 ± 0.45	8.76 ± 0.29	9.56 ± 1.69	10.51 ± 0.32
20 : 1*n*-9	0.40 ± 0.02	0.41 ± 0.04	0.38 ± 0.01	0.45 ± 0.22	0.33 ± 0.00
20 : 4*n*-6 (ARA)	3.09 ± 0.26	3.45 ± 0.11	3.37 ± 0.09	3.11 ± 0.15	3.30 ± 0.07
20 : 5*n*-3 (EPA)	4.07 ± 0.26^b^	8.50 ± 0.29^ab^	10.54 ± 0.11^ab^	11.47 ± 1.25^ab^	13.55 ± 0.25^a^
22 : 6*n*-3 (DHA)	4.81 ± 1.64^b^	4.51 ± 0.28^b^	5.11 ± 0.22^b^	5.73 ± 0.27^b^	8.02 ± 0.19^a^
ARA/EPA	0.76 ± 0.04^a^	0.41 ± 0.01^a^	0.32 ± 0.01^a^	0.27 ± 0.04^a^	0.24 ± 0.01^a^
DHA/EPA	0.95 ± 0.03^a^	0.53 ± 0.02^a^	0.49 ± 0.02^a^	0.50 ± 0.06^a^	0.59 ± 0.02^a^
DHA/ARA	1.56 ± 0.53^a^	1.31 ± 0.09^a^	1.52 ± 0.06^a^	1.85 ± 0.13^a^	2.43 ± 0.06^a^
OA/DHA	4.34 ± 1.86^a^	4.26 ± 0.18^a^	3.77 ± 0.17^a^	3.01 ± 0.42^a^	1.68 ± 0.05^a^
OA/*n*-3 LC-HUFA	1.37 ± 0.63^a^	1.11 ± 0.01^a^	0.96 ± 0.02^a^	0.79 ± 0.09^a^	0.51 ± 0.01^a^
*n*-3/*n*-6	2.52 ± 0.72^a^	2.53 ± 0.05^a^	2.95 ± 0.03^a^	3.36 ± 0.46^a^	3.88 ± 0.12^a^

*Note*: Proximate and FA analysis data represent means ± SD, (*n* = 3). Different superscripts within each row indicate a significant difference between EPA emulsions (ANOVA (*P* ≤ 0.05); Tukey's HSD).

## Data Availability

Data used to support the findings of this study are available upon request.

## Supplementary Materials

Supplementary 1 Graphic summary that synthesizes the contents of the main results of the study in an illustrated and concise way.

Supplementary 2 Larval rearing sequence and sampling points utilized in the study.
