# Supplementary material for: Optimizing Artemia Enrichment: A Low DHA/High EPA Protocol for Enhanced n-3 LC-HUFA Levels to Support Greater Amberjack (Seriola dumerili) Larval Rearing
Source: Aquac Nutr. 2023 Sep 19;2023:5548991. doi: 10.1155/2023/5548991 (PMC10773596; doi:10.1155/2023/5548991)
Supplement: Supplementary 1 — Graphic summary that synthesizes the contents of the main results of the study in an illustrated and concise way. [file 5548991.f1.pdf]

Optimizing Artemia Enrichment: A Low DHA/High EPA Protocol for Enhanced n-3 LC-HUFA Levels to Support Greater Amberjack (Seriola dumerili) Larval Rearing.

This study suggests that EPA-rich oil can be used as an alternative to DHA oil for improving the nutritional value of Artemia as food for greater amberjack larvae and avoid retroconversion

Most commercial Artemia enrichment products with high DHA levels have limited success. This study evaluated the use of EPA rich oil to improve LC-PUFA in Artemia for greater amberjack larvae.

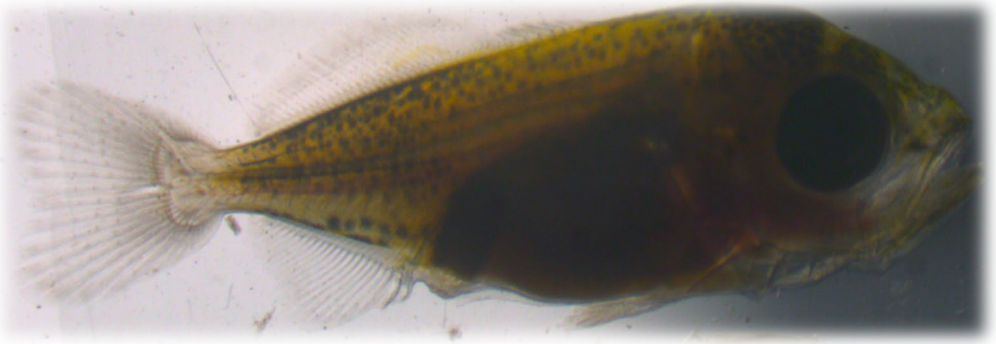

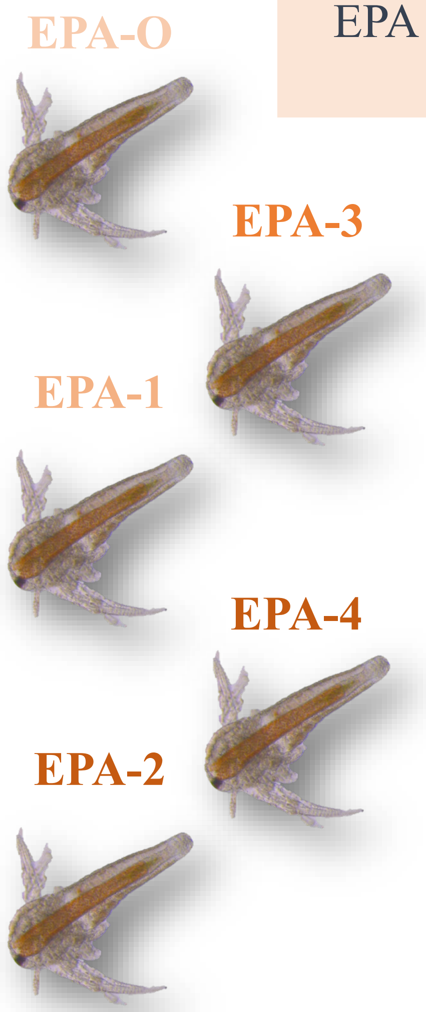

Five experimental emulsions with increasing levels of EPA and n-3 LC-HUFA were fed to larvae in three replicate tanks.

- Use of EPA-rich oils is effective for enhancing nutritional quality of Artemia
- Enrichment with n-3 LC-HUFA content between 11-21% allows for successful growth, survival, and stress resistance of greater amberjack larvae
- Incorporating EPA-rich oils instead of DHA rich oil is preferable due to reduced cost and elimination of retro-conversion of DHA to EPA.

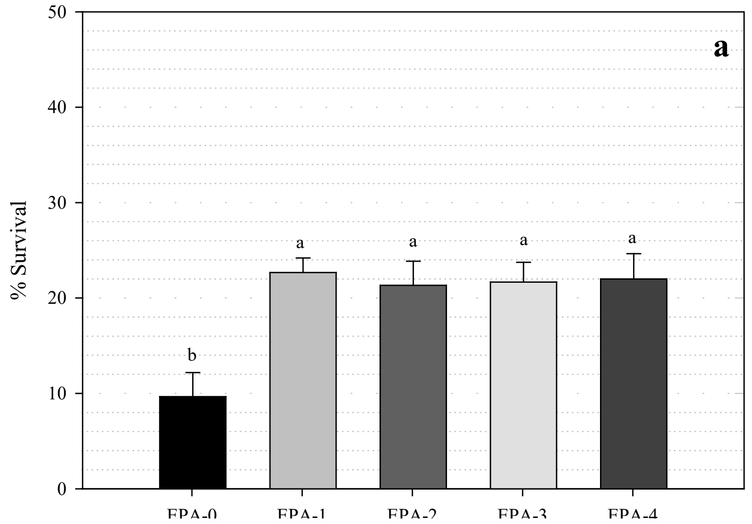

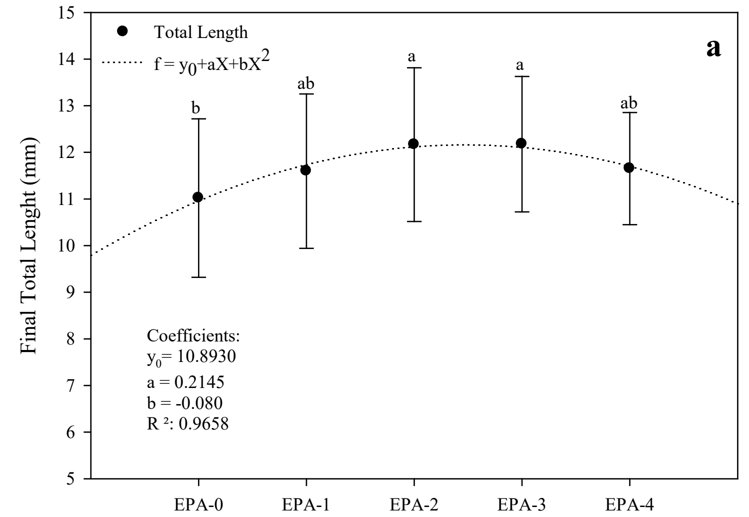

WILEY | Hindawi Aquaculture Nutrition

Roo et al. Aquaculture Research Group (GIA), Institute of Sustainable Aquaculture and Marine Ecosystems (IU-ECOAQUA), Universidad de Las Palmas de Gran Canaria (ULPGC), Carretera de Taliarte, s/n, 35214 Telde, Spain

This was funded by DIVERSIFY project (GA603121) and forms part of the ThinkInAzul programme supported by Ministerio de Ciencia e Innovación with funding from European Union NextGenerationEU (PRTR-C17.I1) and the Comunidad Autónoma de Canarias (GOBCAN_ACIISI)
