# Supplementary material for: Optimizing Artemia Enrichment: A Low DHA/High EPA Protocol for Enhanced n-3 LC-HUFA Levels to Support Greater Amberjack (Seriola dumerili) Larval Rearing
Source: Aquac Nutr. 2023 Sep 19;2023:5548991. doi: 10.1155/2023/5548991 (PMC10773596; doi:10.1155/2023/5548991)
Supplement: Supplementary 2 — Larval rearing sequence and sampling points utilized in the study. [file 5548991.f2.pdf]

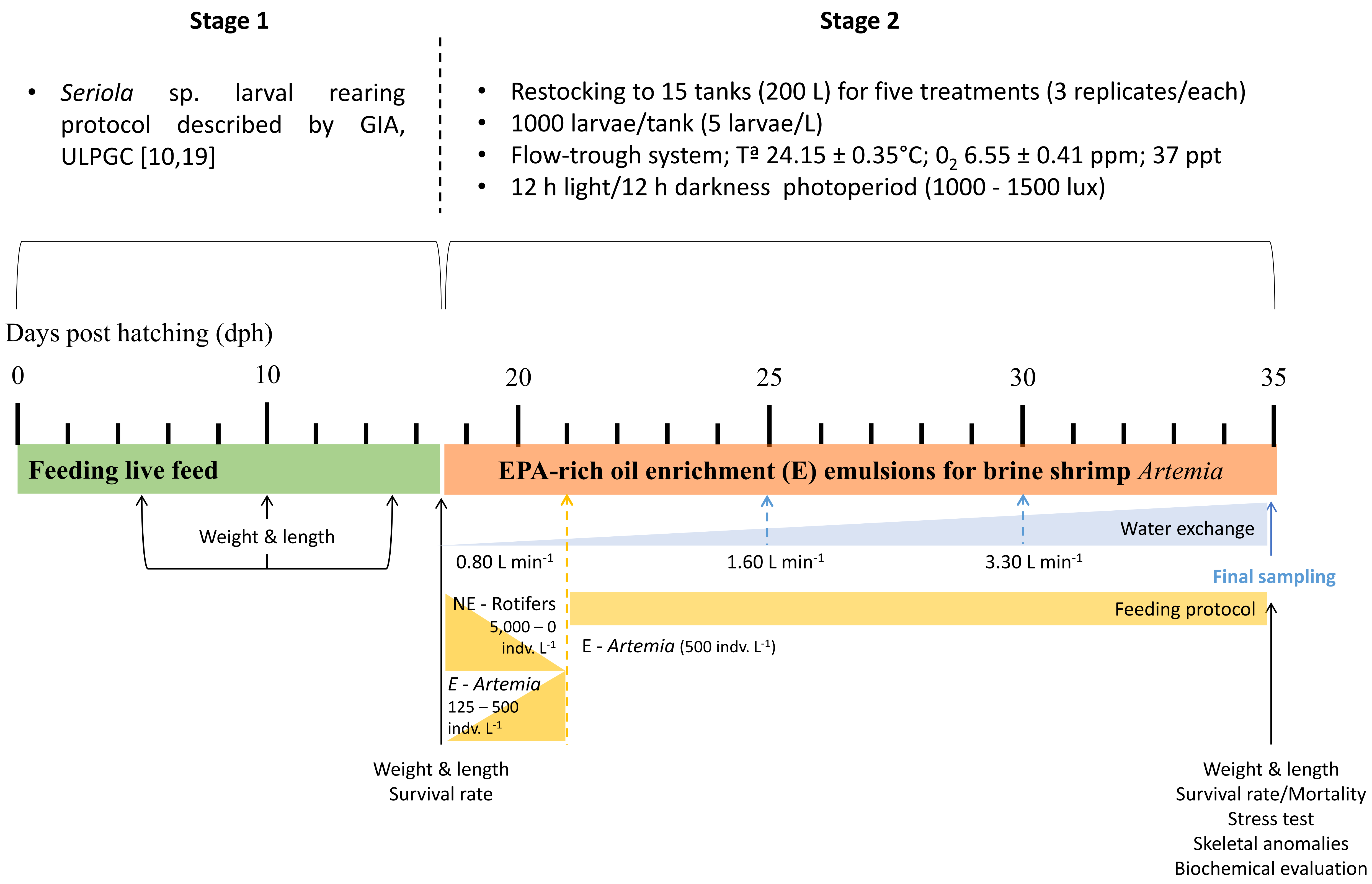
Stage 1
Stage 2
• Seriola sp. larval rearing protocol described by GIA, ULPGC [10,19]
• Restocking to 15 tanks (200 L) for five treatments (3 replicates/each)
• 1000 larvae/tank (5 larvae/L)
• Flow-trough system; Tª 24.15 ± 0.35°C; 0_2 6.55 ± 0.41 ppm; 37 ppt
• 12 h light/12 h darkness photoperiod (1000 - 1500 lux)
Days post hatching (dph)
0
10
20
25
30
35
Feeding live feed
EPA-rich oil enrichment (E) emulsions for brine shrimp Artemia
Weight & length
Water exchange
0.80 L min^{-1}
1.60 L min^{-1}
3.30 L min^{-1}
Final sampling
NE - Rotifers
5,000 – 0
indv. L^{-1}
Feeding protocol
E - Artemia (500 indv. L^{-1})
E - Artemia
125 – 500
indv. L^{-1}
Weight & length
Survival rate
Weight & length
Survival rate/Mortality
Stress test
Skeletal anomalies
Biochemical evaluation
